# A Rare case of central nervous system actinomycosis presenting with moyamoya syndrome

**DOI:** 10.1111/cns.13842

**Published:** 2022-04-11

**Authors:** Min Zhang, Shujuan Meng, Mohammad al mahmoud, Ye Li, Yuwei Dai, Chunhui Li, Jinxia Zhou, Bo Xiao, Lili Long

**Affiliations:** ^1^ Department of Neurology Xiangya Hospital Central South University Changsha China; ^2^ National Clinical Research Center for Geriatric Disorders Xiangya Hospital Central South University Changsha China; ^3^ Infection Control Center Xiangya Hospital Central South University Changsha China

**Keywords:** actinomycosis israelii, CNS actinomycosis, microbiological next‐generation sequencing, moyamoya syndrome

## CONFLICT OF INTEREST

All authors report no disclosures.

## AUTHOR CONTRIBUTION

M.Z. and L.L. conceptualized the study, conducted literature review, interpreted the data, and drafted and revised the manuscript for intellectual content. S.M., M.A.M., Y. D., Y.L., C.L., J.Z., and B.X. interpreted the data and revised the manuscript for intellectual content.

Dear Editor

Central nervous system (CNS) actinomycosis is a rare infectious disease associated with several types of lesions, including abscesses, meningitis/meningoencephalitis, and actinomycetoma.^1^ Here, we report a case of moyamoya syndrome caused by CNS actinomycosis in a woman who had just undergone a cesarean section a month ago and was probably immunocompromised.

## 1 CASE PRESENTATION

**FIGURE 1:**
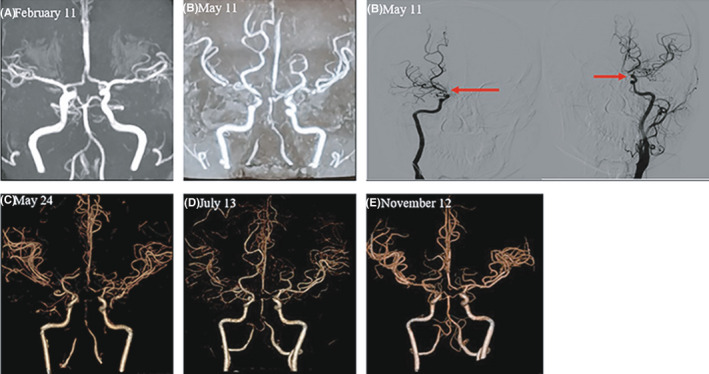
Cerebral vascular imaging before and after this onset. (A) MRA before this onset was normal. (B) MRA on the 10th day of the onset without effective antibiotic therapy indicated the bilateral varying vascular stenosis and occlusion at the beginning of internal carotid artery and middle cerebral artery, posterior cerebral artery, and adjacent collateral blood vessel formation; digital subtraction angiography (DSA) indicated multiple localized stenosis in the intracranial segment of bilateral internal carotid artery, bilateral middle cerebral artery M1 segment, and the initial segment of the basilar artery. (C) CTA on the 24th day of the onset indicated the same vascular stenosis to b. (D) CTA after the 2 months of effective anti‐infective treatment showed the improvement of vascular stenosis. (E) CTA after half a year of effective anti‐infective treatment showed obvious vascular improvement and collateral circulation formation

We report the case of a 30‐year‐old woman admitted to the local hospital for a 10‐day paroxysmal numbness in the left limb, weakness in the left lower limb, accompanying limb tremors, and slow response, on May 11, 2021. Magnetic resonance imaging (MRI) showed multiple acute or subacute infarctions in the bilateral basal ganglia and right frontal lobe and multiple localized stenoses of the circle of Willis (Figure 1B). Cerebral digital subtraction angiography (DSA) revealed moyamoya vascular changes (Figure 1B). An initial diagnosis of moyamoya syndrome was made. Anticoagulation therapy was administered. The patient was discharged without any significant improvement on May 18, 2021.

On May 20, 2021, the patient was transferred to Xiangya Hospital of Central South University. The patient developed persistent weakness in her left limb, although paroxysmal numbness had subsided. Limb tremors and slow reactions were approximately the same as before. Throughout the course, the patient had no fever, headache, vomiting, or other CNS symptoms.

**FIGURE 2:**
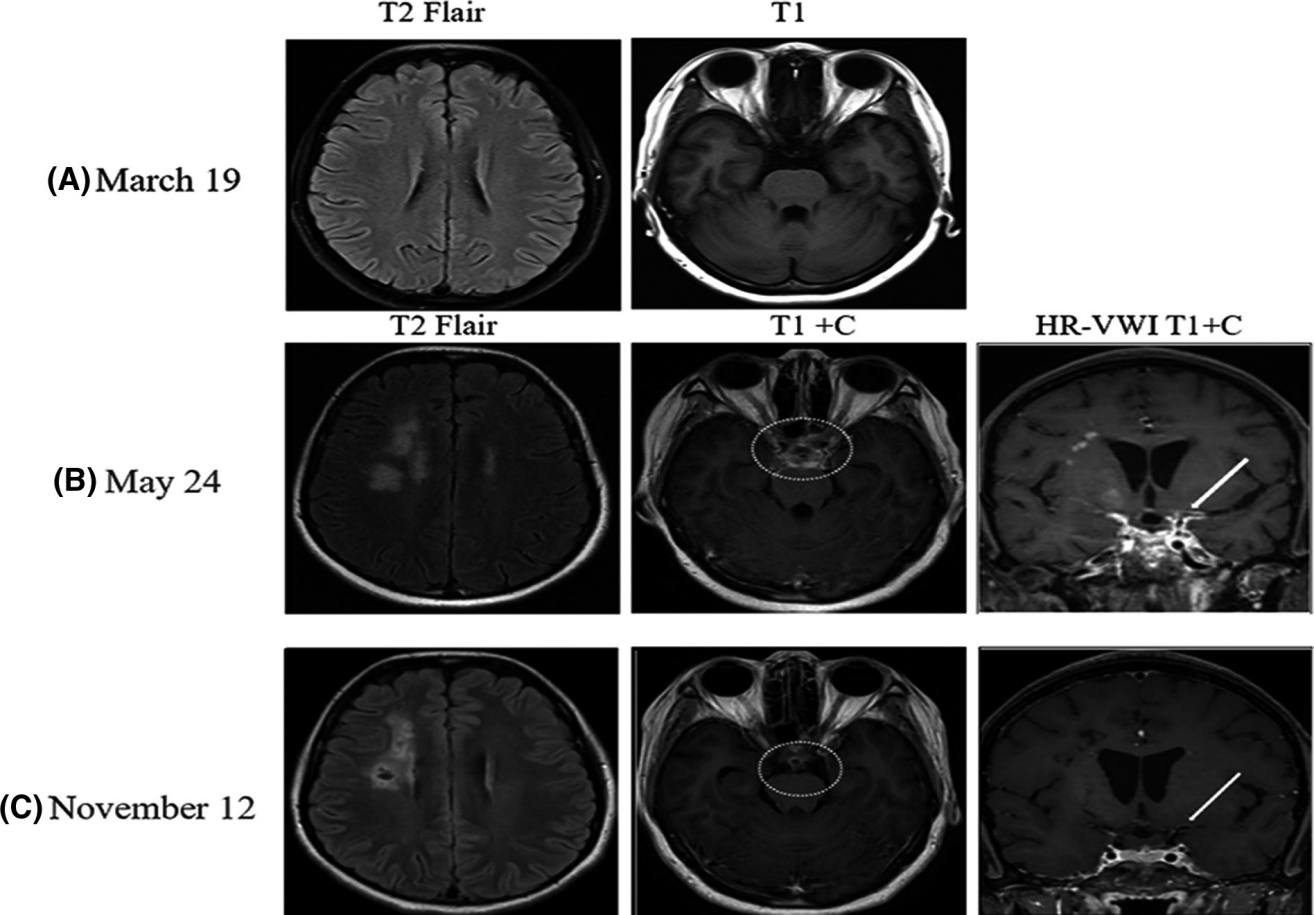
Brain imaging of the patient. (A) MRI before the onset showed normal. (B) MRI on the 24th day of the onset without effective antibiotic therapy showed the acute cerebral infarctions in the right basal ganglia and frontal lobe and meningeal enhancement in slope, saddle cistern, and interfoot cistern. (C) Follow‐up MRI showed marked improvement after the effective anti‐infective treatment (T1+Contrast showed the significantly improved meningeal enhancement, and HR‐VWI T1+C showed the enhancement of vascular wall after improved meningeal enhancement)

Three months ago, the patient presented with a headache and blurred vision was diagnosed with cerebral venous sinus thrombosis at our hospital. Brain magnetic resonance venography (MRV) showed an invisible left transverse sinus and sigmoid sinus, whereas magnetic resonance angiography (MRA) (Figure 1A) and CSF analysis were normal. After treatment with low‐molecular‐weight heparin for 1 month, a repeat brain MRI (Figure 2A) and MRV showed normal results. Rivaroxaban was continued for 3 months to consolidate the treatment of sinus thrombosis. One month before the onset of cerebral infarction, she underwent a cesarean section, gave birth to a healthy baby, and had no puerperal infection. The patient had no history of sinusitis, tooth extraction, head trauma, or other surgical procedures.

On physical examination after admission, the patient showed a slow reaction and speech speed. A slightly shallower left nasolabial fold and drooping of the left corner of the mouth were also present. The power of the left limb was 4/5. The deep tendon reflexes of her limb were active, and a positive Hoffmann sign on the left and bilateral ankle clonus were observed. Nuchal rigidity was present. All other systemic physical examination results were normal. Brain MRI showed the evolution of the cerebral infarction area compared with the onset (Figure 2B) and signs of meningeal enhancement on the base of the skull. Computed tomography angiography (CTA) revealed vascular stenosis or occlusion of the anterior and posterior circulation (Figure 1C). High‐resolution vessel wall imaging (HR‐VWI) revealed irregular thickening of multiple vascular walls (Figure 2B). Serum inflammatory and immunological tests were negative. A lumbar puncture was performed because of the presence of nuchal rigidity and signs of meningeal enhancement. The pressure of the CSF was 220 mmH_2_O, and the color was yellow. CSF analysis revealed 320 WBCs/mm^3^ (75% polynuclear cells), a protein level of 3200 mg/dl, a reduced glucose level of 1.28 mmol/L, and a reduced chloride level of 111.1 mmol/L. Special staining (gram, acid‐fast, and ink) and culture results (bacteria, fungi, and tuberculosis) were negative. CSF samples were collected for metagenomic next‐generation sequencing (mNGS). In total, 148 Actinomyces israelii sequencing reads were detected. These findings indicated meningoencephalitis caused by Actinomyces israelii. We prescribed her intravenous penicillin (240wu q6h) and oral doxycycline (0.1 g bid). With dynamic review and regular anti‐infective treatment, the patients’ symptoms and CSF and imaging features (Figure 1D,E, Figure 2C) were significantly improved.

The clinical course and CSF evolution of the patient are shown in (Figure S1).

## 2 METHODS

The CSF sample from the patient was sequenced using mNGS technology, and the pathogen was confirmed by PCR and Sanger sequencing. Written informed consent was obtained from the patient.

## 3 RESULTS

A total of 148 sequence reads of Actinomyces israelii were detected in the CSF and exclusively corresponded to the genome. The sequencing reads had high coverage in the Actinomyces genome and accounted for approximately 8.97% of the total microbial reads (Figure S2).

## 4 DISCUSSION

In this case, the patient presented with cerebrovascular events without fever, headache, or other central nervous system infection symptoms. Subsequently, she was diagnosed with secondary moyamoya syndrome due to CNS actinomycosis. To the best of our knowledge, this is the first report of CNS actinomycosis causing moyamoya syndrome.

Human actinomycosis is a rare inflammatory disease mainly caused by Actinomyces israelii, one of the anaerobic Gram‐positive bacteria, which normally colonizes the oropharynx, gastrointestinal tract, and urogenital tract.^2^, ^3^ CNS actinomycosis is a rare form of human actinomycosis and accounts for less than 5% of cases but has high mortality; more than half of survivors have neurological sequelae.^1^ Symptoms of CNS actinomycosis are nonspecific and diverse and can mimic malignancy or tuberculosis abscesses.^1^ The lesions associated with CNS actinomycosis include brain abscess, meningitis/meningoencephalitis, actinomycetoma, subdural empyema, and spinal and epidural abscess.^1^, ^2^ Meningitis is the most common form of CNS actinomycosis, except for abscesses. Vascular events are known complications of meningitis. Vascular events associated with meningitis caused by Actinomyces israelii are uncommon, including bilateral cavernous sinus syndrome,^4^ intracranial mycotic aneurysm,^5^ and subarachnoid hemorrhage due to secondary necrotizing arteritis.^6^ However, moyamoya syndrome caused by Actinomyces israelii has not yet been reported. The spread of inflammation of the meninges at the base of the skull may invade the adjacent vasculature, cause secondary vasculitis, and mimic moyamoya vascular changes. CNS actinomycosis is mostly sensitive to beta‐lactams, especially penicillin G and amoxicillin, and requires long‐term anti‐infection treatment.^3^

In the past, the diagnosis of CNS actinomycosis depended more on histopathological examination but less on positive CSF culture, as the result of insufficient CSF culture technology and improper sample transportation.^1^, ^2^ Moreover, a pathological biopsy cannot be performed in most cases. In the case of negative CSF culture and inadvisable pathological biopsy in our patient, we used mNGS to detect 148 sequence reads of Actinomyces israelii in the CSF. Considering the patients’ clinical characteristics, images, CSF tests, the results of mNGS and Sanger sequencing, and the effect of corresponding treatment, we thought that Actinomyces israelii was the pathogenic bacterium in this case. Overall, mNGS plays a crucial role in the diagnosis of CNS infections, but it has certain false‐positive and false‐negative rates. Thus, larger sample studies are required to determine how many sequence reads in CSF are meaningful, and further Sanger sequencing could help to verify the accuracy. In clinical neurology, there is a growing body of literature reporting that mNGS can assist in the diagnosis and treatment of both infectious and non‐infectious brain disorders.^7^, ^8^, ^9^ However, a comprehensive analysis of the combination of clinical presentations, laboratory tests, and follow‐up is needed for a final accurate diagnosis.

## Supporting information

Fig S1‐S2

## Data Availability

The Data of this manuscript is available at http://ngdc.cncb.ac.cn, reference number PRJCA009027.
