# Supplementary material for: A Rare case of central nervous system actinomycosis presenting with moyamoya syndrome
Source: CNS Neurosci Ther. 2022 Apr 11;28(7):1139–42. doi: 10.1111/cns.13842 (PMC9160446; doi:10.1111/cns.13842)
Supplement: Supplementary file 1 — Fig S1‐S2 [file CNS-28-1139-s001.docx]

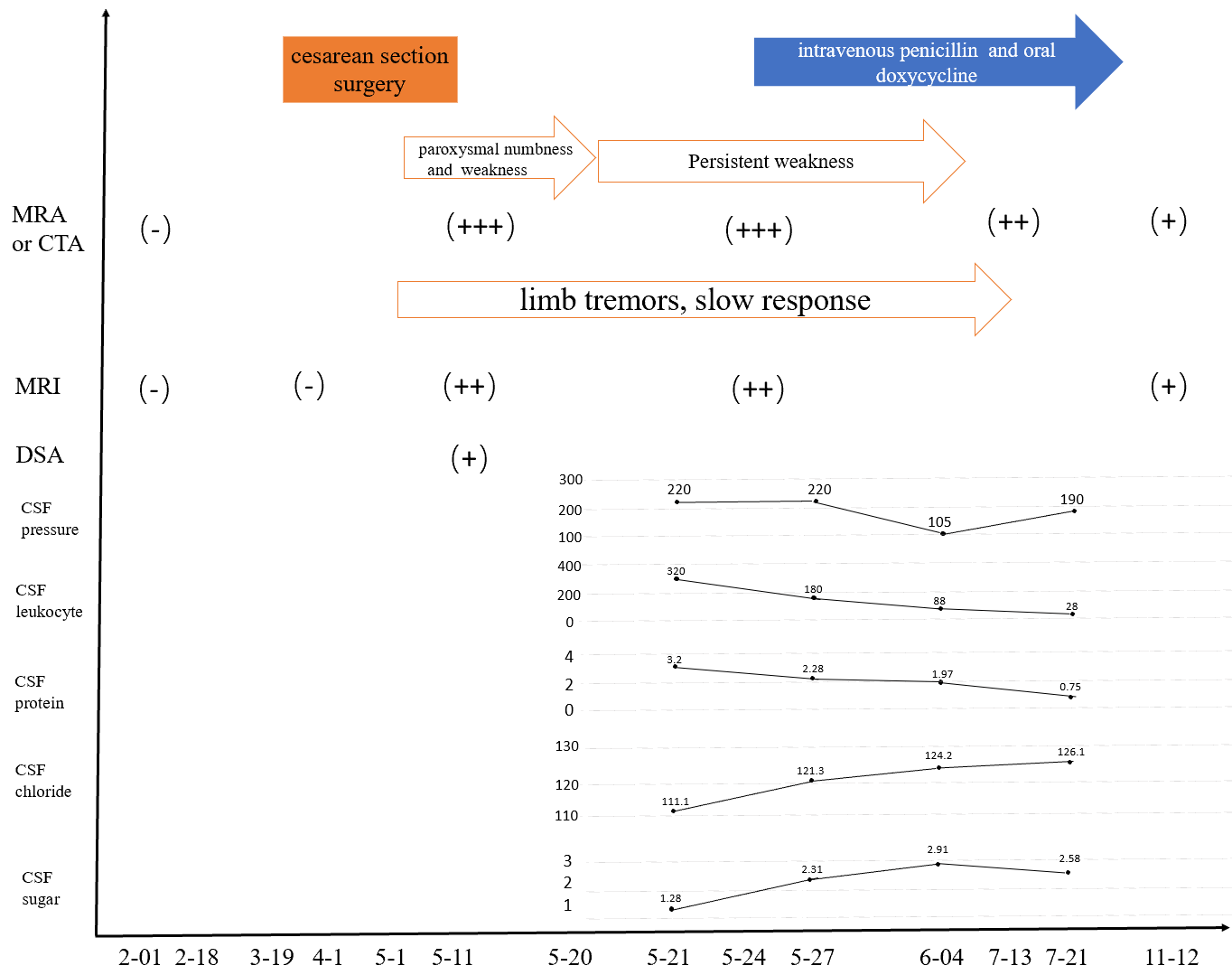


**Supplementary figure 1** The clinical course and CSF evolutions of the patient.


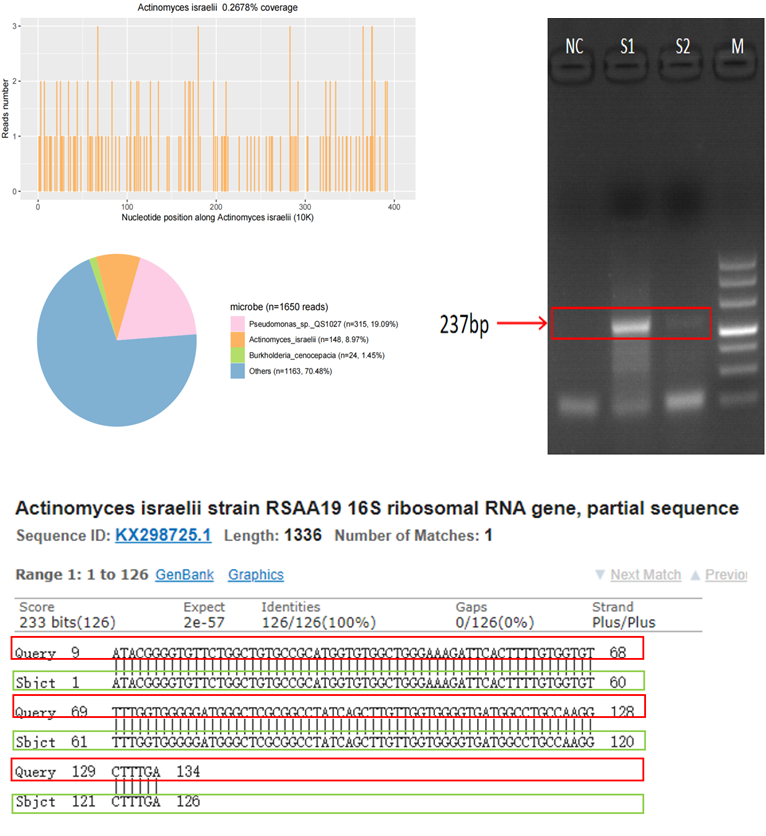


**Supplementary figure 2** Detection of Actinomyces israelii in CSF using the mNGS and following verification using PCR and Sanger sequencing. CSF was collected and transmitted on dry ice for PACEseq mNGS detection (Hugobiotech, Beijing, China). QIAamp DNA Micro Kit (QIAGEN, Germany) was applied to extract DNA following the manufacturer’s instructions. DNA libraries were then constructed using QIAseq™ Ultralow Input Library Kit for Illumina (QIAGEN, Germany) according to its manual. The quality of the constructed libraries was assessed by Qubit (Thermo Fisher) and Agilent 2100 Bioanalyzer (Agilent Technologies). The qualified DNA libraries were finally sequenced on Nextseq 550 platform (Illumina). A total of 148 specific reads of Actinomyces israelii were detected and had a high coverage in actinomyces genome and took up the account of 8.97% of total microbial reads. The 16S rRNA region of this pathogen was then amplified by Nested PCR using primers of F1 5’-AAGTCGAACGGGTCTGCCTTG-3’, F2 5’-TAACCTGCCCCTCACTTCTGG-3’, and R1 5’-TCAAAGCCTTGGCAGGCCATC-3’. The fragments length was 237bp. After agarose gel electrophoresis and Sanger sequencing, Actinomyces israelii was also detected, which confirmed the mNGS result.
